# Separation of Antibiotics Using Two Commercial Nanofiltration Membranes—Experimental Study and Modelling

**DOI:** 10.3390/membranes14120248

**Published:** 2024-11-23

**Authors:** Obinna Anike, Jiří Cuhorka, Nkechi Ezeogu, Petr Mikulášek

**Affiliations:** Institute of Environmental and Chemical Engineering, Faculty of Chemical Technology, University of Pardubice, Studentská 573, 532 10 Pardubice, Czech Republic; obinna.anike@student.upce.cz (O.A.); nkechi.ezeogu@student.upce.cz (N.E.); petr.mikulasek@upce.cz (P.M.)

**Keywords:** nanofiltration, rejection, antibiotics, SHP model, Spiegler–Kedem model

## Abstract

The widespread use of antimicrobial drugs has contributed to the increasing trace levels of contaminants in the environment, posing an environmental problem and a challenge to modern-day medicine seeking advanced solutions. Nanofiltration is one such breakthrough solution for the selective removal of antibiotics from wastewater due to their high efficiency, scalability, and versatility. This study examines the separation of antibiotics (sulfamethoxazole (SMX), trimethoprim (TMP), and metformin (MET), respectively) using commercially available membranes with an emphasis on AFC membranes (AFC 30 and AFC 80). Thus, we evaluate their efficacy, performance, and applicability in wastewater treatment processes. The data for characterizing the structural parameters of the NF membranes were determined from an uncharged organic solute rejection experiment, and the effect of various operating conditions on the retention of solutes was evaluated. All experimental data were collected using a laboratory-scale nanofiltration unit and HPLC, and rejection percentages were determined using analytical measurements. The results obtained allowed for the determination of the radius of the membrane pores using the Steric Hindrance Pore (SHP) model, resulting in values of 0.353 and 0.268 nm for the AFC 30 and AFC 80 membranes, respectively. Additionally, higher transmembrane pressure and feed flow were observed to lead to an increased rejection of antibiotics. AFC 30 demonstrated a rejection of 94% for SMX, 87% for TMP, and 87% for MET, while AFC 80 exhibited a rejection of 99.5% for SMX, 97.5% for TMP, and 98% for MET. The sieving effect appears to be the primary separation mechanism for AFC 30, as lower feed-flow rates were observed to intensify concentration polarization, thereby compromising rejection efficiency. On the contrary, AFC 80 experienced less concentration polarization due to its smaller pore sizes, effectively preventing pore clogging. Membrane performance was evaluated using the Spiegler–Kedem–Katchalsky model, based on irreversible thermodynamics, which effectively explained the mechanism of solute transport of antibiotics through the AFC 30 and AFC 80 membranes in the NF process.

## 1. Introduction

The 20th century is considered a fundamental moment in the history of medicine and a significant advancement in the development of antibiotics, indicating a new era in the prevention of widespread mortality and disease plagues, forever revolutionizing infective disease treatment [1]. However, the 19th century laid the essential groundwork for discovering and developing antibiotics, with the groundbreaking works of scientists like Pasteur, Lister, Koch, and Ehrlich establishing the principles of microbiology and infectious disease treatment. These foundational discoveries and theories set the stage for Alexander Fleming’s eventual finding of penicillin in 1928, which revolutionized medicine and ushered in the modern era of antibiotics [2,3]. The evolution of antibiotics is a testament to the relentless development of scientific knowledge and the profound impact of these discoveries on human health and healthcare in general.

**Figure 1:**
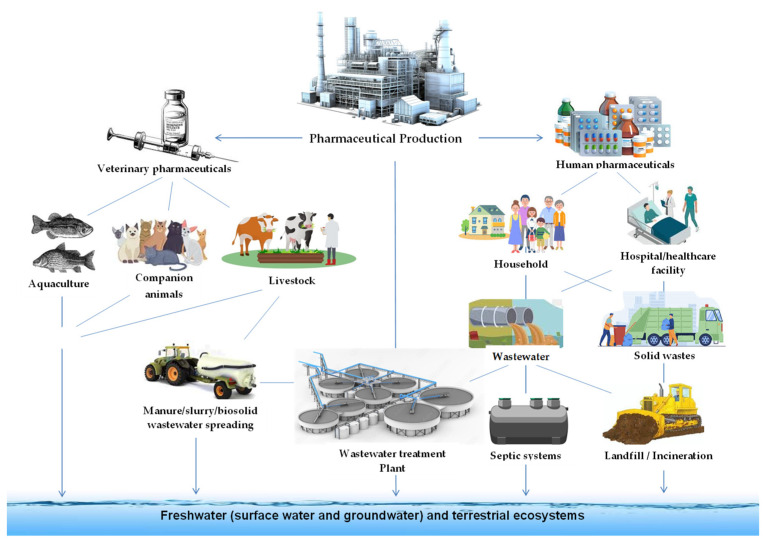
Release of pharmaceuticals into the environment [8].

Today, the use of antibiotics extends beyond therapeutic purposes in various sectors, including animal husbandry, beekeeping, aquaculture, ethanol production, horticulture, antifouling paints, food preservation, and even domestic applications [4]. This contributes to the increasing trace levels of this class of pharmaceutical compounds as contaminants in surface and groundwater. Findings by Klein et al. [5] show that global ingesting increased in 2018, with a staggering intake estimate of “40 billion defined daily doses (DDD) (95% confidence interval: 37 billion–433 billion DDD)” [5]. This represents a significant increase of 46% since 2000, as reported by Browne et al. [6]. A similar report by Bombaywala [7] reveals that an estimated 1 million to 2 million tons of antibiotics are consumed annually. Therefore, a significant amount of antibiotics is expected to be discharged into the environment as most of these antibiotics ingested by humans or animals undergo incomplete metabolism within their bodies [8]. Antibiotics enter the environment through several pathways, as shown in Figure 1, mainly through misuse, overuse, and discharge of untreated industrial effluent [9].

The emergence of antibiotic-resistant diseases, also known as antimicrobial-resistant infections (AMR), is a direct consequence of the prevalent use of antibiotics, which has become a challenge for modern-day medicine [7]. Although resistance to treatment by lethal pathogens is not a new phenomenon, the real challenge lies in the rapidly increasing rate of resistance, which has become alarming. It is predicted that, by 2050, this trend could lead to a significant rise in mortality rates [10,11]. Therefore, it becomes paramount to find feasible steps to mitigate this growing global threat and reduce the risk of infections, severe ailments, and mortality. Membrane-based separation technologies have emerged as promising tools for the selective removal of antibiotics from wastewater due to their high rejection efficiency, energy efficiency, scalability, versatility, and lack of the need for additional chemical treatment [12,13,14,15]. Its application goes beyond wastewater treatment, water desalination, biotechnology, and pharmaceuticals, extending into the food industry, as well as dairy and vegetable oil processing, fruit juice production, plant extracts, and beverage industries [13,16,17]. Several studies have demonstrated the efficacy of NF membranes in the removal of pharmaceuticals, especially commercially available membranes, which offer practical solutions to this pressing environmental challenge, providing a range of membrane types and configurations tailored to specific separation requirements. Numerous commercial NF membranes are used in water treatment processes, each with unique properties such as surface charge, hydrophobicity, pore size, and molecular weight cut-off (MWCO) [12,17,18].

Two widely studied commercial NF membranes are the NF90 and NF 270 membranes, manufactured by Dow FilmTec. These membranes differ in pore sizes and surface charge, leading to varying performance in the removal of organic pollutants, including antibiotics. NF90 membrane has demonstrated tremendous performance as published by several researchers over a decade, especially for rejecting/retaining pharmaceutical active compounds. For instance, López–Muñoz et. al. [19] investigated the NF90 membrane’s ability to remove sulfamethoxazole among other PhACs from wastewater, achieving over 95% rejection. Similarly, Dolar et al. [20] reported rejection rates exceeding 99% for antibiotics like sulfamethoxazole and trimethoprim when using NF90 membranes. Yangali–Quintanilla et al. [21] further showed that NF90′s rejection mechanism is primarily governed by steric hindrance, particularly effective for hydrophilic, neutral compounds. However, NF90′s rejection efficiency can vary under specific conditions. For example, the presence of divalent cations may reduce its performance, and it tends to operate optimally in neutral solutions [21]. In contrast, AFC membranes are known for their high rejection rates, with pore sizes close to those of reverse osmosis (RO) membranes. This makes them especially versatile and suited to extreme pH conditions and high-solid-content effluents. They are chemically stable and resistant to fouling, which makes them effective for industrial applications, even in complex feed waters [9]. A comparison of antibiotic removal is presented in Table 1. AFC membranes are gradually gaining popularity among researchers for the treatment of pharmaceutical-polluted water due to their high flux rates, selective solute rejection properties, and good chemical stability. AFC 80 has a tighter pore size and higher rejection of divalent salts and organic molecules, making it suitable for applications requiring high selectivity. In contrast, the AFC 30 and AFC 40 membranes exhibit a more porous structure, which enhances their permeability to monovalent salts and smaller organic molecules. This characteristic makes them particularly suitable for applications that require a higher permeate flux. The physicochemical properties of the antibiotics can influence the performance of these membranes in the separation of antibiotics, and such factors include molecular size, hydrophobicity, and charge [22,23].

This study focuses on the separation of metformin, sulfamethoxazole, and trimethoprim using commercially available AFC membranes (AFC 30 and AFC 80) to assess their efficacy, performance characteristics, and applicability in wastewater treatment processes. Although not classified as an antibiotic, metformin has an antimicrobial/antibacterial impact on a variation of microorganisms and exerts antimicrobial effects on bacterial strains [24,25,26]. There is a diverse array of commercially available membranes, including microfiltration, ultrafiltration, nanofiltration, and reverse osmosis membranes, which present a spectrum of options for antibiotic removal, each with unique pore sizes, surface properties, and separation mechanisms. By systematically investigating the performance of nanofiltration membranes in antibiotic separation, this research seeks to elucidate the underlying mechanisms governing their effectiveness and to identify optimal membrane configurations for drugs with antimicrobial effects in wastewater. The study was conducted with a higher concentration of drugs and can be used as a precursor for a preliminary screening test for real wastewater that would require sophisticated analyses and expenditure. The concentrations tested, ranging from 5 to 20 mg L^−1^, were selected to examine how antibiotics interact with the chosen membranes and to address the challenges of antibiotic separation in wastewater. Therefore, this is essential to develop sustainable wastewater treatment strategies, as factors such as antibiotic properties, fouling of the membrane, feed concentration, pH, and hydraulic conditions are keys to determining separation efficiency and membrane performance.

**Table 1:** Comparison of antibiotic removal between previous studies and this study in terms of rejection.

Solute	Membrane	Manufacturer	Feed Water	Rejection Max. (%)	Reference
SMX	NF	Dow–Filmtec	Milli-Q water, pH = 6.12–6.67, 10 mg L^−1^ at 15 bar	29.4	Dolar et al. [20]
	NF90	Dow–Filmtec	Milli-Q water, pH = 7, mg L^−1^ at 12 bar	>95	López–Muñoz et al. [19]
	NF90	Dow–Filmtec	Milli-Q water, pH = 6.12–6.67, 10 mg L^−1^ at 15 bar	97.2	Dolar et al. [20]
	NF 270	Dow–Filmtec	Milli-Q water, pH = 7, 10 mg L^−1^ at 12 bar	>75	López–Muñoz et al. [19]
	NF 270	Dow–Dupont	pH = 6.8–7.2, 2 mM at 10 bar	92	Osorio et al. [27]
	NF 270	Dow–Filmtec	Milli-Q water, pH = 6.12–6.67, 10 mg L^−1^ at 15 bar	15.4	Dolar et al. [20]
	Desal HL	GE Osmonics	Syn water, pH 6.5, at 4.2 bar	>80	Wang et al. [28]
	Desal HL	GE Osmonics	Milli-Q water, pH = 6.12–6.67, 10 mg L^−1^ at 15 bar	24.7	Dolar et al. [20]
	AFC 30	PCI membranes	Demi. water, 15 mg L^−1^, 15–30 bar	94	Present work
	AFC 80	PCI membranes	Demi. water, 15 mg L^−1^, 15–30 bar	99.5	Present work
TMP	NF	Dow–Filmtec	Milli-Q water, pH = 6.12–6.67, 10 mg L^−1^ at 15 bar	92.9	Dolar et al. [20]
	NF90	Dow–Filmtec	Milli-Q water, pH = 6.12–6.67, 10 mg L^−1^ at 15 bar	>99.9	Dolar et al. [20]
	NF 270	Dow-Filmtec	Milli-Q water, pH = 6.12–6.67, 10 mg L^−1^ at 15 bar	86.9	Dolar et al. [20]
	Desal HL	GE Osmonics	Syn water, pH 6.5, at 4.2 bar	80	Wang et al. [28]
	Desal HL	GE Osmonics	Milli-Q water, pH = 6.12–6.67, 10 mg L^−1^ at 15 bar	78	Dolar et al. [20]
	AFC 30	PCI membranes	Demi. water, 3 mg L ^−1^, 15–30 bar	87	Present work
	AFC 80	PCI membranes	Demi. water, 3 mg L^−1^, 15–30 bar	97.5	Present work
	DK	GE Osmonics	surface water, pH 6.37, 35.99 ng L^−1^ at 10 bar	94	Foureaux et al. [29]
MET	NF99	Dow Chemical	Syn water pH = 3–10, 10 mg L^−1^ at 10 bar	70	Hidalgo et al. [30]
	NF 270	Dow–Dupont	pH = 6.8–7.2, 2 mM at 10 bar	82	Osorio et al. [27]
	AFC 30	PCI membranes	Demi. water, 20 mg L^−1^, 15–30 bar	87	Present work
	AFC 80	PCI membranes	Demi. water, 20 mg L^−1^, 15–30 bar	98	Present work

## 2. Materials and Methods

### 2.1. Nanofiltration Membranes and Antibiotics

The tubular-designed NF membranes (AFC 30 and AFC 80) manufactured by PCI Membranes Systems, Kostrzyn Poland, were employed. According to the manufacturer’s specifications, both membranes are designed for operation at temperatures below 60 °C, with a maximum operating pressure of 60 bar, and within a pH range spanning from 1.5 to 10.5, as specified in Table 2. These membranes are manufactured using the thin-film composite (TFC) technique with an aromatic polyamide surface layer on a porous polysulfone substrate. AFC 30 and AFC 80 exhibit nominal rejection rates of CaCl_2_ at 80% and 75%, respectively.

All drug standards (sulfamethoxazole (SMX), trimethoprim (TMP), and metformin (MET)) of high purity grades (>98%) were obtained from Sigma Aldrich, St. Louis, MO, USA. The drug tablets used are Biseptol 400 mg/80 mg (sulfamethoxazole/trimethoprim) produced by Adamed Pharma S.A, Pieńków, Poland and Siofor 1000 (Metformin) produced by Laboratori Guidotti S.p.A, Pisa, Italy were all purchased from Dr Max Pharma, Praha, Czech Republic. Table 3 shows the physiological data of the chemicals and drugs used.

### 2.2. Nanofiltration Setup and Experiments

**Figure 2:**
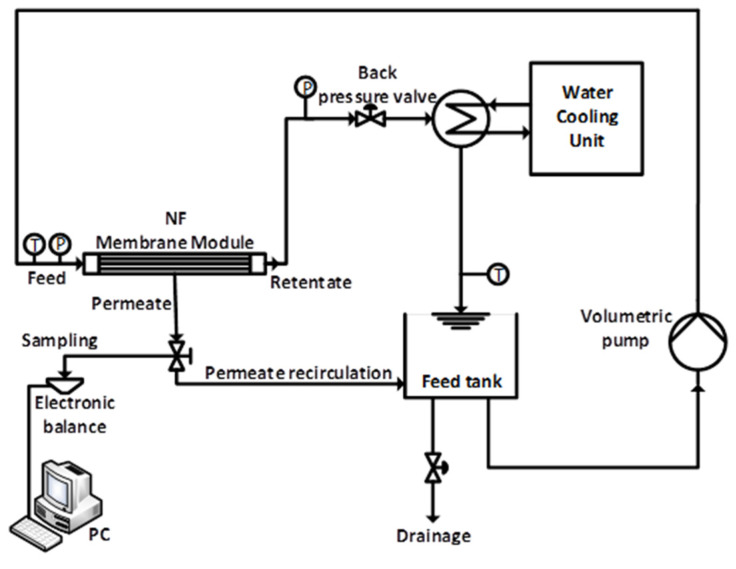
Schematic diagram of the nanofiltration set-up.

Figure 2 presents a cross-flow-designed separation unit used for this experiment [35]. It adopts a batch circulation mode in which both the retentate and permeate are reverted to the feed tank to ensure that there are no feed concentration differences. The permeate flux was determined with software installed on a personal computer and connected to an electronic scale balance (Balance KERN KB, Kern and Sohn GmbH, Balingen, Germany). The software uses the weight of permeate collected over a specified time interval. The specified membrane’s effective surface area and permeate density are used to determine the flux of the demineralised water according to Equation (1). Samples of permeate are collected at different transmembrane pressures after sufficient recirculation to ensure constant permeate concentration and flux. Preliminary concentration measurements of the permeate reveal that after recirculation of 800 mL of permeates, the system achieves a steady state.

Before using the membranes to perform NF separation experiments, we prepared the membranes to ensure stable and optimum performance by conducting two major procedures. First, the membranes were conditioned by immersion in distilled water for at least 72 h. The conditioned membranes were then loaded into the module of the NF unit and compacted with distilled water by gradually increasing the transmembrane pressure from 5 bar to 30 bar at a stable temperature of 25 ± 0.5 °C controlled by a water cooling unit (TAEevo, Armfield, GB, Ringwood, UK), and then held at 31 bar within a time frame of at least 2 h. Demineralized water was pumped at 15 L min^−1^ flow rate to reduce the greater chances of concentration polarization [35]. At the end of the procedure, the distilled water used for the compaction test was discharged, and the membrane was stored with fresh distilled water for 48 h before the start of the water flux and drug-rejection experiments. This procedure was crucial to ensure the stable performance of the membrane without undergoing any permanent deformation or damage that can impact its performance during separation while maintaining the membrane quality and performance standard.

The water flux experiment was performed at various pressure differences (5–30 bar) to assess membrane permeability. Uncharged solute rejection was measured to determine the average membrane pore size using a feed solution containing 500 mg L^−1^ of the organic solute (glycerol, glucose, or isopropyl alcohol). The membrane was soaked in the feed solution for 72 h before the first drug experiment due to possible adsorption of drug on the membrane. This minimizes the effect of sorption on separation efficiency. Then, drug rejection and flux under varying process conditions (feed concentration, feed flow rate, and ionic strength) [41] were measured.

### 2.3. Analytical Procedures

The SPE-HPLC analytical method was adopted to determine the permeate concentrations [42]. This technique is often applied to concentrations that are below the limits of detection (LOD) and quantification (LOQ) for HPLC. The solid-phase extraction (SPE) technique was primarily used for the preparation, purification, and concentration of the permeate prior to HPLC investigation. In this case, the HPLC sensitivity was increased and enhanced, as the TMP was observed to be far below the detection limit during HPLC analysis. Agilent 1260 Infinity II LC System (HPLC instrument), Santa Clara, CA, USA, was equipped with two binary pumps (1260 Infinity II flexipump G7104C, Agilent technologies, Santa Clara, CA, USA) that operate up to 800 bar, a diode array detector, DAD (1260 Infinity II WR G7115A), fluorescence detector spectra, FLD (1260 Infinity II G7121B, Agilent technologies, Santa Clara, CA, USA), and multisampler (1260 Infinity II G7167A, Agilent technologies, Santa Clara, CA, USA) was used for this analysis. Chromatographic separation was successful using the C18 (5 µm, 250 mm × 4 mm) Nucleosil 120 column (Macherey-Nagel GmbH & Co., Dueren, Germany) with a mobile phase in isocratic condition. The analytical methods were similar to previously published work for the simultaneous determination of SMX, TMP, and MET [43,44]. HPLC conditions are displayed in Table 4.

### 2.4. Membrane Performance

Accessing the performance of both NF membranes in rejecting selective solutes (pharmaceutical compounds) is determined from experimental data of three fundamental factors.

Flux (*J*) is calculated from Equation (1) and defined as the ratio of permeate volume flow across the membrane (*V* in m^3^) to the total surface area of the membrane (*A* in m^2^) at the particular time (*t* in s)

(1) $$J=\frac{V\;}{A\times t}$$

Rejection (*R*), also known as observed rejection (*R_o_*), is calculated as the ratio of the concentration difference between the feed (*C_f_*) and the permeate (*C_p_*) to the feed concentration (*C_f_*) as expressed as a percentage as in Equation (2):

(2) $$R(\%)=(1-\frac{Cp\;}{Cf})\times 100$$

However, this rejection does not reflect the true rejection of the membrane as a result of an increase in the accumulation of solute at the membrane surface. Therefore, a concentration build-up resulting in diffusive flow back, as such, opposes the convective solute flow towards the surface of the membrane. The membrane surface concentration (*C_i,m_*) differs entirely from that of the bulk retentate (*C_i,f_*). Therefore, to determine the real solute rejection, also referred to as intrinsic rejection (*R_int_*), the film model is used to calculate the concentration at the membrane surface (*C_i,m_*), as in Equation (3), and is employed for solute real rejection in Equation (4), or directly as the last part of Equation (4):

(3) $$C_{i,m}=C_{i,p}+\left(C_{i,f}-C_{i,p}\right)exp\left(\frac{J}{k}\right)$$

(4) $$R_{int}=1-\frac{C_{i,p}}{C_{i,m}}=\frac{R_{o}\times exp\left(\frac{J}{K}\right)}{1-R_{o}\left[1-exp\left(\frac{J}{K}\right)\right]}$$

where J is the permeate flux determined experimentally from the permeate volume (*V_p_*) collected over the effective surface area (*A*) in a time difference (Δ*t*). *K* is the mass transfer coefficient calculated using the Sherwood relationship for the tubular membrane and the turbulent flow regime (Deissler correlation) provided in Equation (5).

(5) $$Sh=\frac{Kd_{h}}{D_{i\infty }}=0.023\times {Re}^{0.875}\times {Sc}^{0.25}$$

The Sherwood, Reynolds, and Schmidt numbers are denoted as *Sh*, *Re*, and *Sc*, respectively. $D_{i\infty }$ is the diffusivity of the solute at infinite dilution (m^2^ s^−1^), while *d_h_* represents the hydraulic diameter of the membrane (m).

### 2.5. Predictive Models for Nanofiltration Membranes

#### 2.5.1. Steric Hindrance Pore Model (SHP)

The SHP model was employed to investigate the structural characteristics of the membranes used. Although it was initially developed to separate aqueous solutions of single organic solutes in ultrafiltration membranes, Wang et al. [40] successfully applied it to characterize NF membranes [40]. It is a predictive model with two adjustable parameters σ and ω, as provided in Equations (6) and (7), to estimate structural parameters of membranes, such as pore radius and porosity-to-thickness ratio:

(6) $$\sigma_{SHP}=1-S_{F}\left\{1+\left(\frac{16}{9}\right)q^{2}\right\}$$

(7) $$\omega_{SHP}=D\times S_{D}\left(\frac{A_{k}}{\Delta x}\right)$$

(8) $$where\;S_{D}={\left(1-q\right)}^{2}$$

(9) $$S_{F}=2{\left(1-q\right)}^{2}-{\left(1-q\right)}^{4}$$

(10) $$q=\left(\frac{r_{s}}{r_{p}}\right)$$

In the equations above, $\frac{A_{k}}{\Delta x}$ signifies the relationship between the porosity of the membrane and its thickness, while *D* is diffusivity at infinite dilution. The terms *r_s_* and *r_p_* denote the Stokes and pore radius, respectively. Additionally, the hindrance factors for diffusion and convection were represented as *S_D_* and *S_F_* respectively.

#### 2.5.2. Spiegler–Kedem–Katchalsky (SKK) Model

The simplicity of the SKK model makes it a better choice for researchers to gain first insight into solute transport through membranes with charged surfaces, as employed in this study. It should be noted that since the charge effect affected transport mechanism, a new type of transport (electromigration) can be involved. This type of transport is omitted for the SKK model. The model is based on principles of non-equilibrium thermodynamics and characterizes the transport of solutes through the membrane, which it depicts as a black box system [47]. Consequently, membranes are characterized using this model with three key transport coefficients, pure water permeability (*L_p_*), solute permeability (*ω_s_*), and reflection coefficient (*σ*), to describe solute rejection and the permeability of water and solute through the membrane [35]. These membrane coefficients are related to the solute flux (*J_s_*) and the water flux (*J_w_*), according to Equations (11) and (12).

(11) $$J_{s}=\omega_{s}\cdot {\Delta C}_{s}+\left(1-\sigma \right){\cdot J}_{v}\cdot C_{i,m}$$

(12) $$J_{W}=L_{p}\left(\Delta P-\sigma \Delta \pi \right)$$

where *C_i,m_* remains as defined in Equation (4), Δ*C_s_* denotes the concentration difference between the permeate and the membrane surface, and the difference in osmotic pressure difference between both fluids is provided as Δ*π*. The values of these coefficients are determined by fitting the Spiegler–Kedem Equation (13) to the experimental data on the rejection-versus-flux plot for σ and ω_s_. The permeability of water (*L_p_*) is determined from the dependency of the flux-versus-pressure difference (ΔP) data.

(13) $$R_{int}=\sigma \cdot \left(\frac{1-F}{1-\sigma \cdot F}\right)$$

(14) $$\mathrm{Where}\;F=exp\left(-\frac{1-\sigma }{\omega_{s}}\cdot J_{v}\right)$$

The model is validated using the non-linear factor $x^{2}$, as presented by Equation (15), which serves as a metric to evaluate the fit quality of the models:

(15) $$x^{2}=\sum \frac{{\left(R_{int}-R_{SKM}\right)}^{2}}{R_{SKM}}$$

## 3. Results and Discussion

Table 5 highlights individual experiments performed on each nanofiltration membrane used in this study. It indicated that not all effects were measured and presented especially for AFC 30 due to insignificant results (isopropyl alcohol, similar trend of concentration polarization effect for other solutes) or fouling (limiting experiments for AFC 30 membrane). The study recommends the AFC 80 membrane with a lower irreversible fouling tendency compared to AFC 30.

### 3.1. Demineralized Water Permeability

**Figure 3:**
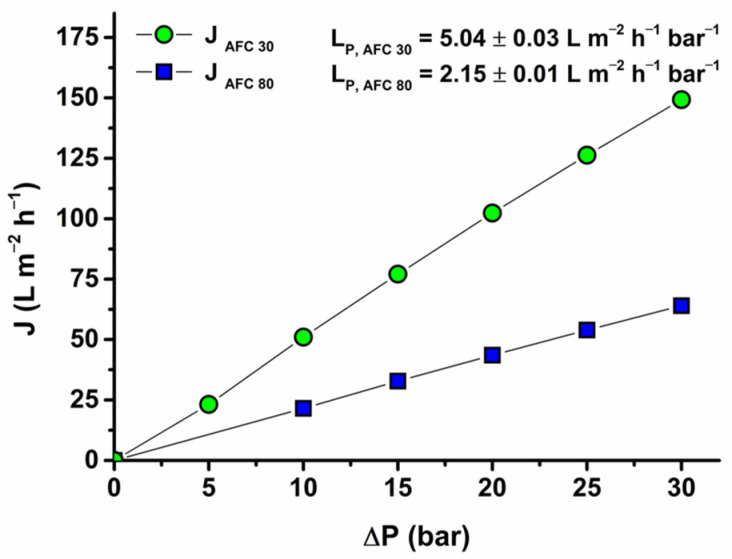
Influence of operating pressure for the AFC 30 and AFC 80 membranes on water flux.

After the compaction test, a water permeability test was performed as a preliminary step in understanding the structure of the membranes. After the compaction test, a water permeability test was performed as a preliminary step to understand the structure of the membranes. The result is presented in Figure 3, which shows a clear dependence of the water flux on the operating pressure for both membranes. The relationship is linear as expected and as reported by other researchers. However, AFC 80 twice recorded a lower water permeability (2.15 L m^−2^ h^−1^ bar^−1^) than AFC 30 (5.04 L m^−2^ h^−1^ bar^−1^), and this suggests an evident distinction in its structural properties (pore size, void space, and material). AFC 80 must have been designed for higher resistance with a smaller pore size, while AFC 30 is designed for higher throughput with a more porous structure. These assumptions agree with the value of their pore sizes calculated using the SHP model in Section 3.2.

### 3.2. SHP Model and Determination of Structural Parameters

The retention measurement of uncharged, organic solutes in aqueous solution, and the water permeability was used to assess the pore dimension of the membranes (AFC 30 and AFC 80). The results were analysed and fitted with the SHP model to determine the key components presented in Table 6. The average radius of the membrane pore based on the rejection of two uncharged solutes, glucose (Glu) and glycerol (Gly) for AFC 30, and isopropyl alcohol (IPA) and glycerol for AFC 80, were 0.353 and 0.268 nm, respectively. These uncharged organic solutes were selected on the basis of the demineralised permeability flux data to suggest possible dense or porous membranes, their molecular weights to provide information on size selectivity, and diffusivity to provide insights into how the membrane influences solute transport. The typical general pore size of NF membranes ranges between 0.3–2 nm, as reported by several authors and researchers. A more specific range of 0.4 to 1.5 nm, with a majority within 0.40–0.8 nm for commercial membranes like AFC membranes [40,48,49], was observed. However, some are at the lower end of nanofiltration pore sizes, such as the AFC 80, approaching the range more typical for reverse osmosis (RO) membranes. For instance, Adeniyi et al. [50] classified AFC 80 as an RO membrane due to its aperture size. Furthermore, Bruggen and Vandecasteele [51] worked with three membranes (NF70, UTC-20, and NTR 7450) and reported their average pore sizes, respectively, as 0.34, 0.54, and 0.80 nm—a standard deviation parameter sp of 0.54, 0.17, and 0.52 nm, respectively. Bowen and Doneva [52] published an AFM quantification of pore-size distribution among other characteristics of AFC 30, AFC 80, and XDA9920 membranes from PCI membranes. The results revealed a mean pore radius of 0.44, 0.34, and 0.58 nm. Recent works reporting aperture sizes of AFC 80 membranes as 0.38 (±0.24) nm [53], 0.283 nm [9], and 0.262 nm [41] reveal similar pore sizes within the range of our result. This also confirms the suggestions in Section 3.1 that AFC 30 is a loose NF membrane when compared with AFC 80, which is considered a dense membrane with much smaller pores.

The average reflection coefficient (σ) for both membranes tends toward 1.0 (excluding Gly on AFC 30), indicating a high rejection for selected solutes.

### 3.3. Evaluation Performance of AFC 30 and AFC 80 NF Membranes

#### 3.3.1. Influence of Operating Pressure

**Figure 4:**
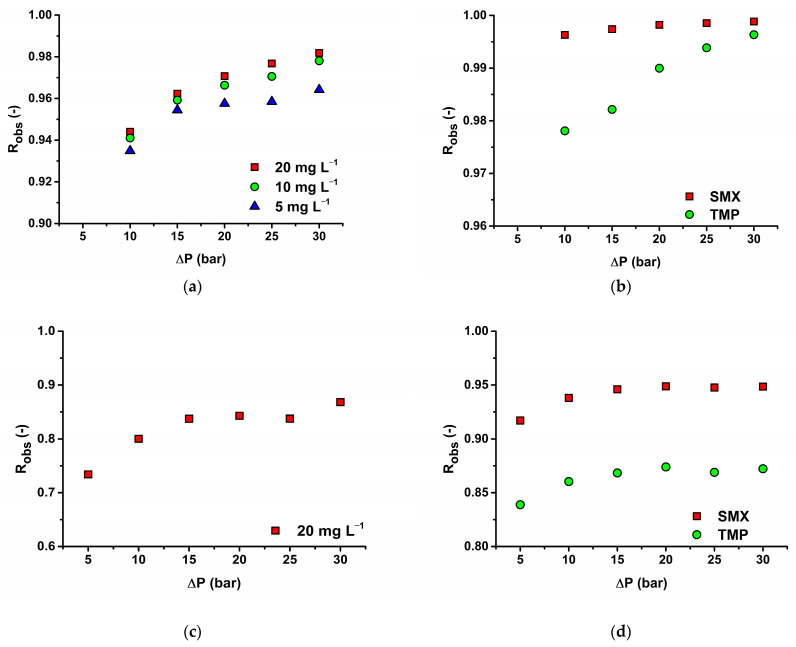
Influence of operating pressure at 15 L min^−1^ on (**a**) MET for AFC 80; (**b**) SMX and TMP for AFC 80; (**c**) MET for AFC 80; and (**d**) SMX and TMP for AFC 30.

The effect of operating pressure was first studied to see the performance of the membranes. This evaluation was conducted on MET and a mixture of SMX within a pressure range of 5–30 bar for AFC 30 and 10–30 bar for AFC 80. As the operating pressure increases, the permeate flux through the membrane typically increases due to the increased driving force for solvent movement across the membranes as presented in Figure 4. Giacobbo et al. [14] found that the apparent rejection coefficients for pharmaceutical active compounds (PhAC), including SMX, increased with transmembrane pressure, indicating that higher pressures can lead to improved rejection rates for certain solutes [14]. This trend is consistent with the solution-diffusion model, which posits that while the solvent flux increases with operating pressure, the solute flux remains relatively constant, leading to higher rejection efficiencies [14]. Moreover, the relationship between operating pressure and rejection efficiency might be influenced by concentration polarization, a phenomenon where solutes accumulate at the membrane surface, potentially leading to reduced rejection rates at higher pressures. More about the effect of concentration polarization is included in Section 3.3.3.

SMX and TMP studies for the effect of pressure difference were also concluded for 20 mg L^−1^ of SMX, and the corresponding concentration of TMP (4 mg L^−1^) on the AFC 80 membrane, as well as the results, is shown in Figure 4b. The results for 20 mg L^−1^ were only published due to the inadequacies of the SPE-HPLC technique to detect lower concentrations (5 and 10 mg L^−1^) below LOD and LOQ. From Figure 4b, the rejection of SMX was >99%, and that of TMP was >97% at all pressure differences. The high rejection rate of SMX might be due to the combined electrostatic influence and steric hindrance resulting from the negative charge of SMX and relatively high molecular size, leading to more frequent interference between antibiotic molecules and the membrane surface, thus influencing the selectivity of the membrane and consequently increasing the rejection. This aligns with the observations made by Han et al. [54], who noted that the rejection of various solutes, including antibiotics, is affected by the electrostatic forces between the solute and the membrane surface, which can be modulated by the concentration of the feed solution [54]. However, in the case of TMP, the rejection is more influenced by the pressure difference than for SMX on the AFC 80 membrane.

The rejection of all drugs on the AFC 30 membrane is lower compared to the AFC 80 membrane, which can be attributed mainly to a denser structure (enhanced rejection due to the sieving effect) of the AFC 80 membrane. For example, MET rejection ranges from 73% to 84% for AFC 30. It must be noted that we assume a lower rejection of this smaller solute. Our results may be influenced by membrane fouling. Experiments with MET on this membrane were measured after experiments with a mixture of SMX and TMP and some fouling appeared. More about it will be discussed in Section 3.3.3. Compared to the AFC 80 membrane, the observed rejection on this membrane is more influenced by the applied transmembrane pressure. It can be explained by the higher influence of the convective solute transport for this membrane. The rejections of the tested antibiotic are compared with the results of other commercial membranes in Table 1.

#### 3.3.2. Influence of Concentration on Antibiotic Rejection Efficiency

**Figure 5:**
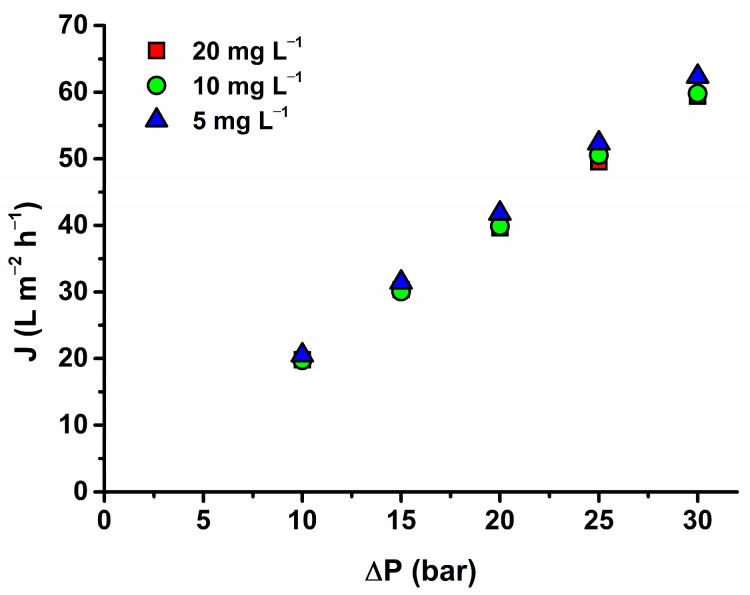
Influence of MET feed concentration on flux at 15 L min^−1^ for AFC 80.

The impact of concentration was studied at a maintained flow rate of 15 L min^−1^ and varying concentrations (5 mg L^−1^–20 mg L^−1^) for MET on the AFC 80 membrane as presented in Figure 4a. The results demonstrate a maximum rejection of about 98% and an increasing observed rejection with increasing concentrations. This trend is supported by the model data in Table 7 where it reveals that the MET rejections decrease (as a parameter σ) with decreasing concentration. It can be seen from the table that the reflection coefficient decreases with decreasing feed concentration of MET. This parameter signifies the theoretical rejection obtained at high flux when diffusive transport can be negligible compared to convective solute transport across the membrane. The reason why the rejection of MET decreased with decreasing feed concentration can be seen in reversible fouling (an assumption of MET adsorption on the membrane wall) and thus in changing membrane structure (not pores), acting as the secondary barrier. This phenomenon can be confirmed by decreasing the flux with increasing MET concentration in the feed (see Figure 5). It must be noted that the flux decrease is minor (approx. 5% between 5 and 20 mg L^−1^), and the flux reached an almost stable value over 10 mg L^−1^. Due to our experimental order, when we consecutively decreased the feed concentration and the flux increased, we can conclude that it was a reversible type of fouling. We hypothesize that MET adsorption on the membrane wall forms a new layer, primarily influencing the diffusion of MET by altering its dissolution in the membrane matrix. Since the permeate flux remains nearly constant with only a minor decrease, convection effects can be considered negligible, and significant pore size changes due to fouling are not assumed. Additionally, solute concentration may affect rejection by modifying the membrane’s charge, thereby altering the dissolution rate of MET. This highlights the interplay of surface interactions and transport mechanisms.

#### 3.3.3. Influence of Feed-Flow Rate on Intrinsic Rejection of Antibiotics

**Figure 6:**
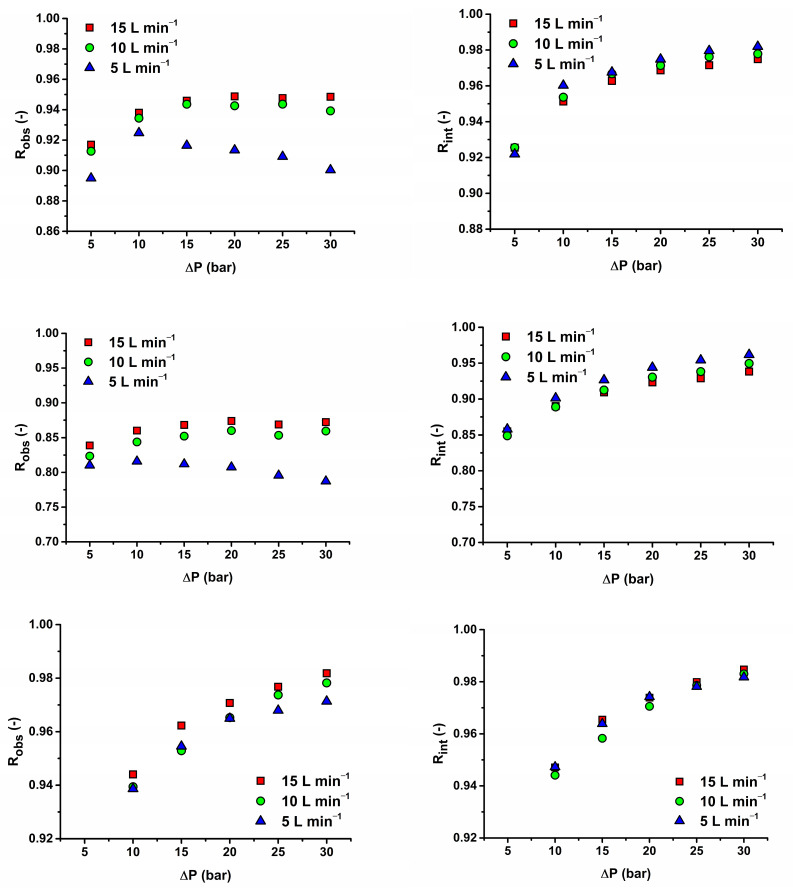
Comparison of observed (left pictures) and real rejection (right pictures) at 15 L min^−1^ for SMX on AFC 30 (first row), TMP on AFC 30 (second row), and MET on AFC 80 (third row).

The flow rate plays an integral part in evaluating the efficiency of membranes in rejecting drugs. Figure 6 demonstrates the influence of the feed flow on the removal efficiency of SMX and TMP by AFC 30 and MET by AFC 80. In this study, the relationship between pharmaceutical rejection and flow rate can be described as complex. The feed-flow rate was studied from 5 to 15 L min^−1^ and resulted in a huge impact on the observed rejection, especially at a lower feed-flow rate (5 L min^−1^), while the intrinsic rejection was completely independent of the flow rate [9]. This is because of concentration polarization mainly for the AFC 30 membrane. However, at a higher feed-flow rate (10 and 15 L min^−1^), the effect of concentration polarization was reduced, although not drastically, due to enhanced turbulence near the surface of the membrane. Beyond pressures of 15 bar at 5 L min^−1^ flow rate for membrane AFC 30, the observed rejection starts to decrease with increasing transmembrane pressure difference for both solutes. For this reason, if the process is restricted to using a feed flow rate of 5 L min^−1^, working at a pressure below 15 bar for an optimal rejection value must be recommended.

In contrast, the rejection efficiency in the AFC 80 membrane for MET witnessed increased observed rejection with each increase in flow rate and pressure. This implies that an increase in the feed-flow rate enhances the mass-transfer coefficient and helps prevent antibiotics from diffusing through the membranes even more. The size of the membrane pore plays a significant role because the pores are not susceptible to concentration polarization. This makes AFC 80 better membranes for separating drugs. It was also discovered that intrinsic rejection followed a similar trend irrespective of the type of membrane, pore size, molecular weight of the pharmaceutical compound, and flow rate.

**Figure 7:**
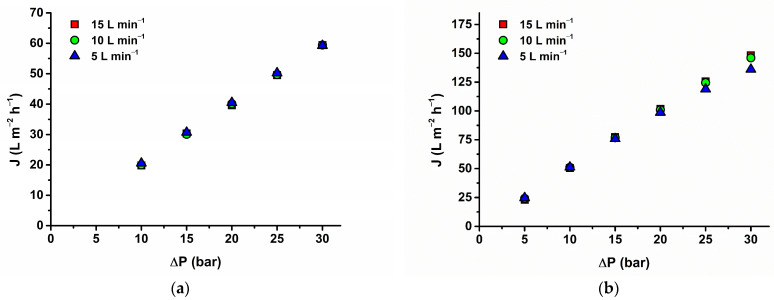
Influence of operating pressure on (**a**) MET for AFC 80 and (**b**) SMX and TMP for AFC 30 on flux for tested feed-flow rates.

Moreover, the influence of the feed-flow rate on the flux must be explored. Figure 7 describes the influence of the feed-flow rate on flux. As can be seen for membrane AFC 80, no effect appeared. This confirmed the results of the experiment when we tested the effect of the feed concentration and found that above 10 mg L^−1^ flux was almost constant. For the AFC 30 membrane, a decrease in flux values can be seen with a decreasing feed-flow rate, mainly for higher pressure differences (above 20 bar). This implies some fouling of the membrane as the osmotic pressure is negligible for the concentration range used. Because the initial permeability of the virgin membrane cannot be restored by flushing with demineralized water, this can be attributed to internal pore blocking (irreversible fouling for physical cleaning). It can influence rejection as a result of a decrease in the pore size. If we look at the intrinsic rejection (Figure 6 right side, first and second row), indeed, intrinsic rejection reached higher values for lower feed-flow rates for AFC 30. In comparison, the intrinsic rejections are without any trend for AFC 80.

### 3.4. Effect of NaCl

**Figure 8:**
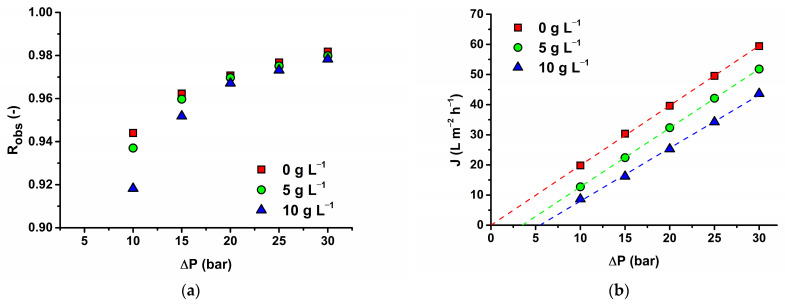
Influence of NaCl at 15 L min^−1^ on (**a**) MET rejection; (**b**) flux for the AFC 80 membrane.

The influence of ionic strength on rejection and flux was studied for MET and AFC 80 membranes because of their relatively smaller molecular weight compared to SMX and TMP. NaCl was used to provide the ionic strength effect needed for this study to understand the combined effects of electrostatic repulsion and size exclusion on membrane performance. The observed rejection decreased with increasing ionic strength (NaCl concentration). This effect is more evident for lower pressure differences (5 and 10 bar). Under this condition, the diffusive flow through the membrane cannot be entirely neglected. Increasing NaCl concentration decreased flux for the same value of applied transmembrane pressure difference and, for this reason, increased the influence of diffusion on solute transport across the membrane. Theoretically, we operate our process (NF) at a lower pressure difference. Figure 8b reveals, as expected, a general decrease in flux with increasing concentration of NaCl due to increased osmotic pressure and, for this reason, a decrease of the net driving force. Another explanation for the decrease in rejection with increasing ionic strength can be the effects of salting out and pore swelling [55]. The first phenomenon decreased hydrated ion size and the second increased the pore size. Decreasing rejection of small positive-charged drugs with increasing concentration can be due to the decrease of electromigration transport [27]. In the SKK model used in our work, this type of transport is omitted.

### 3.5. Antibiotic Rejection Modeling Using the Spiegler–Kedem–Katchalsky (SKK) Model

**Figure 9:**
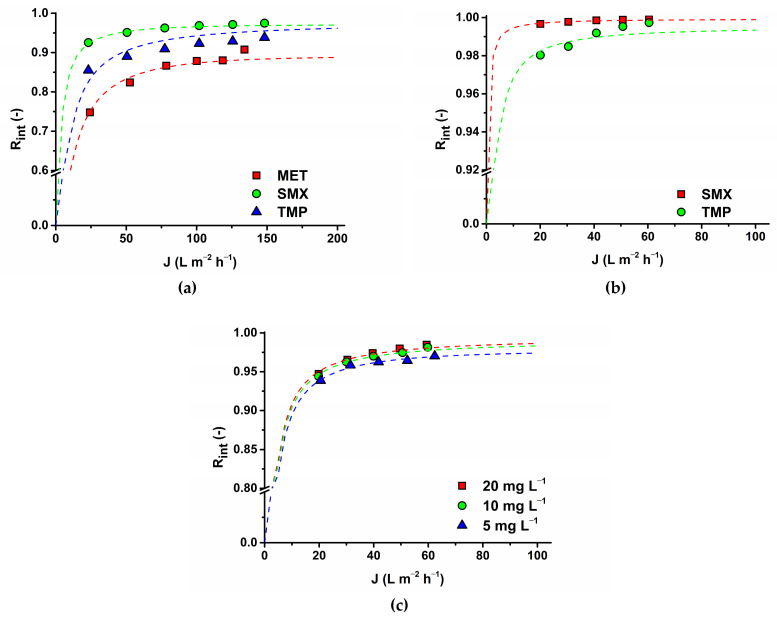
SKK fitting for (**a**) SMX, TMP, and MET on AFC 30; (**b**) SMX and TMP on AFC 80; and (**c**) MET on AFC 80.

With the representation of the Spiegler–Kedem–Katchalsky model as a black box system, it becomes easier to model the rejection of antibiotics by correlating the model with the experimental data provided in Figure 9 and to understand the mechanism of solute transport, with the model parameter shown in Table 8. The data in the table illustrate a high rejection efficiency since the recorded reflection coefficients (σ) tend to unit (0.89–0.999). This implies that the membranes used for the study (AFC 30 and AFC 80) have a high capacity to reject antibiotics, and AFC 80 had the best rejection capacity, which is consistent with previous studies conducted by [9].

The relationship between the intrinsic rejection of SMX, TMP, and MET (*R_Int_*), as well as the model rejection (R_SKM_), indicates an absolute fit shown in the figures for both membranes. The values of χ^2^ in Table 8 confirm the fitting precision of the adjustment in a great way, especially for AFC 80. MET had the least good fitting, as a result of its positive charge and lower molecular weight, thus recording the least rejection. In contrast, SMX had the best rejection owing to its negative charge, thus causing repulsion by the charged surface of the membrane and the influence of steric hindrance due to its large molecular weight. Hence, the applicability of the Spiegler–Kedem–Katchalsky model as a tool for predicting the separation of antibiotics is in alignment with the previous [9,47]. Thus, its usefulness for material selection (membrane), or process conditions for optimum outcomes in wastewater treatment with pharmaceuticals in view, is demonstrated.

## 4. Conclusions

In conclusion, this study demonstrates the efficacy of commercial tubular membranes (AFC 30 and AFC 80) in separating antimicrobial drugs, specifically sulfamethoxazole (SMX), trimethoprim (TMP), and metformin (MET). The primary separation mechanism is the sieving effect, which selectively rejects larger antibiotic molecules over smaller ones and water. Additionally, diffusive transport influences separation, particularly for the AFC 80 membrane.

The pore sizes of the membrane (0.353 nm for AFC 30 and 0.268 nm for AFC 80) are crucial in determining the membrane category and solutes to be retained. The rejection efficiency increased with increasing molecular weight of the antibiotics for positive-charged compound (TMP (87%) > MET (87%) for AFC 30 and TMP (97.5%) > MET (98%) for AFC 80. Notably, SMX, a negatively charged compound, exhibited the highest rejection SMX (94%) for AFC 30 and 99.5% due to charge repulsion with the membrane surface, highlighting the role of charge interference in the separation process.

The study also noted the impact of concentration polarization on rejection efficiency. AFC 30 experienced intensified concentration polarization at lower feed-flow rates, while AFC 80 maintained higher rejection rates due to its smaller pore sizes, which helped reduce clogging. Other investigations on flow rate, ionic strength, and concentrations indicate that AFC 80 is less affected by external influence, thus presenting AFC 80 as a suitable alternative for the most researched dense membrane—NF90.

The performance of the membranes was analyzed using the Spiegler–Kedem–Katchalsky model, providing insights into solute transport mechanisms and factors affecting separation efficiency. Though the study tested the impact of pressure, flow rate, and ionic strength at high drug concentrations, it serves as a preliminary step for selecting suitable membranes. Future research will focus on environmental drug concentrations and long-term assessments of concentration polarization and fouling.

## Figures and Tables

**Table 2:** Characteristics of NF membrane used.

Structural Features	AFC 30	AFC 80
Membrane Type	Tubular	
Material/substrate	Polyamide selective layer/polysulfone	
Max. pressure (bar)	60	
Max. temperature	60	
Surface charge	Negative	
Effective membrane area (cm^2^)	240	
Membrane length (cm)	30	
Internal diameter (cm)	1.25	

**Table 3:** Physiochemical properties of the pharmaceuticals and chemicals used.

Physiochemical Properties	SMX	TMP	MET	Glycerol	Glucose	Isopropyl Alcohol
Charge	Negative ^a,g^	Positive ^g^	Positive ^e,g^	Neutral ^f,g^	Neutral ^j^	Neutral
Diffusion Coefficient (10^−10^ m^2^ s^−1^)	6.12 ^a^	5.6 ^i^	12.3 ^e^	9.5 ^f^	6.7 ^j^	1.02 ^k^
Molecular formula	C_10_H_11_N_3_O_3_S	C_14_H_18_N_4_O_3_	C_4_H_12_ClN_5_	C_3_H_8_O_3_	C_6_H_12_O_6_	C_3_H_8_O
Molecular weight (g mol^−1^)	253.28 ^b,c,d^	290.32 ^c,d^	165.62 ^e,h^	92.09 ^f^	180.16 ^j^	60.1 ^k^
pKa	1.70, 5.7 ± 0.1 ^a,b^	6.8 ^c,d^	12.4^e,h^	14.4 ^f^	12.16 ^j^	12.26 ^k^
Stokes radius (nm)	0.39 ^a^	0.45 ^i^	0.328 ^e^	0.258 ^f^	0.355 ^j^	0.241 ^k^
Structural formula	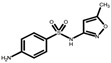	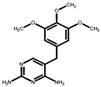	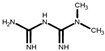	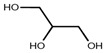	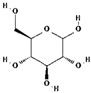	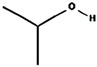

^a^ [31], ^b^ [32], ^c^ [32,33], ^d^ [34], ^e^ [30], ^f^ [35], ^g^ [36], ^h^ [37], ^i^ [38] ^j^ [39], ^k^ [40].

**Table 4:** HPLC parameters for analytical methods.

HPLC Parameters	SMX	TMP	MET
Mobile phase (phosphate buffer: ACN; %)	60:40	60:40	65:35
Conc of phosphate buffer (mM L^−1^)	25	25	15
pH	5.8	5.8	5.8
Retention time (min)	4.5	5.52	2.47
Max. absorbance wavelength (nm)	266	204	236
Water solubility (25 °C) in mg L^−1^	610 ^a^	400 ^b^	1380 ^c^

^a^ [45], ^b^ [46], ^c^ [36], ACN is acetonitrile.

**Table 5:** Summary of the experiments conducted.

Experiments	Test	Membrane	
		AFC 30	AFC 80
Water flux		√	√
Uncharged solutes	Glycerol	√	√
	Glucose	√	
	Isopropyl alcohol		√
MET separation	Effect of pressure difference	√	√
	Effect of feed conc.		√
	Effect of feed flow rate		√
	Effect of ionic strength		√
SMX and TMP separation	Effect of pressure difference	√	√
	Effect of feed-flow rate	√	

**Table 6:** SHP model and membrane structural parameters.

Membrane	Solute	σ	ω	χ^2^	r_p_	Δx/A_k_
	(0)	(-)	(m s^−1^)	(-)	(m)	(m)
AFC 30	Glu	9.82 × 10^−1^	5.47 × 10^−7^	1.59 × 10^−4^	3.77 × 10^−10^	4.19 × 10^−6^
	Gly	8.10 × 10^−1^	9.03 × 10^−6^	5.14 × 10^−3^	3.29 × 10^−10^	4.88 × 10^−6^
AFC 80	Gly	9.86 × 10^−1^	6.82 × 10^−7^	1.32 × 10^−5^	2.72 × 10^−10^	3.64 × 10^−6^
	IPA	9.66 × 10^−1^	7.95 × 10^−7^	1.62 × 10^−4^	2.63 × 10^−10^	8.81 × 10^−6^

**Table 7:** SKM model parameters for AFC 80 and tested MET feed concentration.

C_f_	σ	ω	χ^2^
(mg L^−1^)	(-)	(m s^−1^)	(-)
20	9.93 × 10^−1^	2.66 × 10^−7^	3.96 × 10^−5^
10	9.88 × 10^−1^	2.74 × 10^−7^	2.45 × 10^−5^
5	9.77 × 10^−1^	2.86 × 10^−7^	1.83 × 10^−5^

**Table 8:** SKK model parameter for SMX, TMP, and MET.

Membrane	Solute	σ	ω	χ^2^
		(-)	(m s^−1^)	(-)
AFC 80	MET	9.93 × 10^−1^	2.66 × 10^−7^	3.96 × 10^−5^
	SMX	9.99 × 10^−1^	1.38 × 10^−8^	6.47 × 10^−7^
	TMP	9.94 × 10^−1^	8.18 × 10^−8^	6.71 × 10^−5^
AFC 30	MET	8.90 × 10^−1^	1.59 × 10^−6^	1.02 × 10^−3^
	SMX	9.71 × 10^−1^	4.23 × 10^−7^	7.55 × 10^5^
	TMP	9.72 × 10^−1^	1.22 × 10^−6^	3.00 × 10^−3^

## Data Availability

All data are included in the article.
